# Does interactive ultrasound intervention relieve minor depressive symptoms and increase maternal attachment in pregnancy? A protocol for a randomized controlled trial

**DOI:** 10.1186/s13063-022-06262-4

**Published:** 2022-04-15

**Authors:** Henrika Pulliainen, Sari-Ahlqvist-Björkroth, Eeva Ekholm

**Affiliations:** 1 Department of Clinical Medicine, University of Turku, Turku, Finland; 2 Department of Psychology and Language Pathology, University of Turku, Turku, Finland; 3 Department of Obstetrics and Gynecology, Turku University Hospital and University of Turku, Turku, Finland

**Keywords:** Interactive ultrasound, Pregnancy ultrasound, 4D ultrasound, Perinatal depression, Minor depression, Maternal-fetal attachment, Mother-infant relationship, psychological support

## Abstract

**Background:**

Perinatal depression, especially minor depression, is common during pregnancy and is likely to continue into the postpartum period. It may impair the mother’s health, the infant’s neurodevelopment, and the mother-infant relationship. Screening for perinatal depression is recommended; however, there is no consensus on how to treat depressive symptoms while simultaneously supporting the mother-infant relationship. Ultrasound examination has been shown to improve maternal-fetal attachment among pregnant women. Our aim is to develop a four-dimensional (4D) based interactive ultrasound intervention and test whether it relieves minor depressive symptoms and improves maternal-fetal attachment. Previous studies show that supporting the mother-infant relationship aids in relieving maternal depression. Until now, few studies have combined pregnancy ultrasound and psychological support.

**Methods:**

A controlled randomized setting was designed to assess whether interactive 4D-ultrasound intervention would decrease maternal depressive symptoms, strengthen maternal-fetal attachment, and mother-infant relationship. An obstetrician and a psychologist specialized in infant mental health conduct the interventions. The focus is to jointly observe the behavior of the fetus according to the mothers’ wishes. Altogether, 100 women scoring 10–15 on Edinburgh Pre-/Postnatal Depression Scale (EPDS) and with singleton pregnancy are recruited using a web-based questionnaire. Half of the participants will be randomized to the intervention group and will undergo three interactive ultrasound examinations. The primary outcomes are a decrease in perinatal depressive symptoms assessed with EPDS and an increase in maternal attachment. The maternal attachment was assessed using the Working Model of the Child Interview (WMCI), the Maternal Antenatal Attachment Scale (MAAS), and the Maternal Postnatal Attachment Scale (MPAS). Secondly, we hypothesize that if the intervention decreases prenatal depressive symptoms and improves prenatal attachment, the decrease in depressive symptoms and improvement in mother-infant relationship is seen postnatally.

**Discussion:**

Ultrasound is widely used during pregnancy. The interactive approach is unique and may be feasible as part of routine screenings and maternity clinic visits. Intervention that decreases depression and simultaneously supports maternal-fetal attachment would be a valuable addition to the treatment of minor depression among pregnant women.

**Trial registration:**

ClinicalTrials.gov NCT03424642. Registered on January 5 2018.

## Administrative information

Note: the numbers in curly brackets in this protocol refer to SPIRIT checklist item numbers. The order of the items has been modified to group similar items (see http://www.equator-network.org/reporting-guidelines/spirit-2727-statement-defining-standard-protocol-items-for-clinical-trials/).

**Table:** 

Title {1}	Does interactive ultrasound intervention relieve minor depressive symptoms and increase maternal attachment in pregnancy? – A protocol for a randomized controlled trial
Trial registration {2a and 2b}.	Registered on January 5, 2018, ClinicalTrials.gov NCT03424642
Protocol version {3}	November 3, 2019. Version 4.
Funding {4}	The study is funded by the Signe and Ane Gyllenberg foundation, the Finnish Cultural foundation, the Finnish Medical Foundation and State Research Funding from Hospital Districts of Satakunta and Southwest Finland.
Author details {5a}	Henrika Pulliainen, MD Department of Clinical Medicine, University of Turku, Turku, Finland hpherm@utu.fi Sari Ahlqvist-Björkroth, PhD, Psychology Department of Psychology and Language Pathology, University of Turku, Turku, Finland sarahl@utu.fi Eeva Ekholm, MD, PhD, Adjunct Professor, Obstetrics and Gynecology Department of Obstetrics and Gynecology, Turku University Hospital and University of Turku, Turku, Finland eeva.ekholm@tyks.fi
Name and contact information for the trial sponsor {5b}	Not applicable. This study received no external funding from a sponsor.
Role of sponsor {5c}	Not applicable. This study received no external funding from a sponsor.

## Introduction

### Background and rationale {6a}

Perinatal depressive symptoms are a significant health problem affecting both mothers’ and infants’ well-being. According to systematic reviews, the global prevalence of perinatal depression varies between 8.5 and 11.9% [1, 2]. The incidence in the Finnish population is between 7 and 13% [3–5] and is 14.5% internationally [6]. The use of different instruments and cut-off points to assess depression may explain the varying results [7]. In a Finnish cohort sample, 24% of pregnant women reported moderate levels of depressive symptoms throughout their pregnancy [8]. The prevalence of minor depression during pregnancy has not been studied as systematically as major depression, even though minor depression may be more common [9, 10]. Maternal depressive symptoms during pregnancy tend to remain stable and continue into the postpartum period [1, 11–15].

Maternal perinatal depressive symptoms have an adverse effect on the early mother-infant relationship through three components: pre- and postnatal attachment, mental representations and parent-infant interaction behavior. Maternal prenatal depressive symptoms have a negative association with attachment to the unborn infant [13, 16–19], which, in turn, predicts low maternal involvement in the mother-infant interaction [20, 21]. Prenatal depressive symptoms have also been shown to be risk factors for suboptimal postnatal maternal attachment [13, 22]. It has been found that pregnant women at the most risk of low levels of attachment are depressed and have negative feelings toward the upcoming birth and parenthood [19]. Furthermore, various studies have shown that prenatal depression distorts the mothers’ representation of the infant and their own upcoming motherhood, which, in turn, predicts dysfunctional postnatal mother-child interaction [23–25]. Prenatal representations of pregnant women have been shown to remain quite stable from pregnancy to the postnatal period [26–28]. Maternal depression predisposes the infant to several adverse neurodevelopmental risks [29–31]. A Finnish longitudinal study suggests an additive effect of maternal pre- and postnatal depressive symptoms on child neurodevelopment [32].

The fetal effects of antidepressant medication used during pregnancy should be taken into account. For example, fetal exposure to selective serotonin reuptake inhibitors (SSRIs), which are commonly used antidepressants [33], is associated with neonatal maladaptation [34] and increased rates of depression in early adolescence [35]. The literature has consistently highlighted the importance of identifying women at risk of perinatal depression by screening depressive symptoms and determining effective and individual prevention strategies [11, 25, 36, 37]. However, a decrease in a mother’s depressive symptoms alone does not necessarily improve parenting or the infant’s well-being [38–41]. Some evidence indicates that relationally focused treatments may be more effective than traditional treatments in treating maternal depression [38, 42]. Supporting the mother-infant relationship makes it possible to decrease maternal depression [13, 43, 44]. Thus, early interventions supporting the mother-infant relationship or parenting should be available in primary health care [45, 46] and should start before birth to improve the child’s subsequent development of the child [43, 47].

Ultrasound examinations have a beneficial impact on maternal-fetal attachment [48–51] in low-risk pregnancies. Especially, three-dimensional (3D) and four-dimensional (4D) ultrasounds can enhance mental images about the fetus and increase bonding [52, 53]. There are few studies on combining pregnancy ultrasound and psychological support [54–59]: these interventions are based on an ultrasound consultation method, where the fetus is observed with the mother-initiated interaction [54]. A study involving a small group of women with low-risk pregnancies found that participation in ultrasound consultation could improve maternal attachment and decrease general anxiety symptoms [55]. Intervention in parenting using interactive 4D ultrasound with a focus on mentalization among substance-abusing pregnant women showed high attendance in intervention visits compared with routine care [58]. The way in which ultrasound examinees comment on and interpret the ultrasound images and fetal actions influences parents’ prenatal mental representations of the fetus [60]. Routine ultrasound examinations provide pregnant women a step toward parenthood and a possibility to see the fetus [61] and reduce their worried state of mind [62].

Using interactive ultrasound, we conducted a qualitative study to map the experiences of women at risk of preterm birth. The pregnant women’s experience of the intervention was that it made the fetus more real, strengthening their feelings of attachment to their fetus [59]. The interactive ultrasound intervention has the potential to strengthen maternal prenatal attachment and representations and decrease anxiety and depression. Pregnancy ultrasound may thus be used as a tool for assessing fetal risks, but also an instrument in prenatal psychological interventions.

### Objectives {7}

#### Aim

The primary aim of the present study is to evaluate, whether interactive ultrasound decreases perinatal depressive symptoms by increasing maternal attachment. The secondary aim is to evaluate whether interactive ultrasound improves also postnatal mother-infant interaction.

The specific hypotheses are as follows:

1. The intervention decreases prenatal depressive symptoms and improves the attachment to the fetus.
  - The mothers in the intervention group will have fewer depressive symptoms after intervention than before the intervention.
  - The decrease in depressive symptoms will be greater in the intervention group than in the control group.
  - The improvement of prenatal attachment from the pre-intervention to the post-intervention time will be greater in the intervention group compared with the control group.
  - The mothers in the intervention group will have a more balanced attachment representation after the intervention than before the intervention.
  - The change from non- balanced to balanced attachment representations will be greater in the intervention group than in the control group.
2. If the intervention decreases prenatal depressive symptoms and improves prenatal attachment, the effect prevails postnatally and improves mother-infant interaction.
  - The decrease in the depressive symptoms remains in the intervention group 4 to 5 months old.
  - The mothers in the intervention group will have a stronger attachment to their infant when the child is 4 to 5 months old.
  - The quality of mother-infant interaction will be higher in the intervention group than in the control group when the child is 4 to 5 months old.

Moreover, we will explore the effect of the intervention on various forms of maternal psychological distress. However, too little is known about the trajectories of pregnancy-related anxiety in low-risk populations to state a hypothesis about this. For perinatal outcomes such as preterm birth (< 37 gestational weeks, [gwks]), intrauterine growth restriction (IUGR), and low (< 2.5 kg) birth weight at delivery, the power of the sample is likely to be too low to gain significant differences between groups.

### Trial design {8}

The study design is a randomized controlled trial utilizing an intervention group and a control group. Only the intervention group will participate in three interactive ultrasound examinations. All the data collection measures will be done for both intervention and control groups. Also, the attachment representation interviews about their mental health representation of their unborn baby and relationship with the baby will be done twice during the pregnancy for both groups. The trial design is described in the research chart (Appendix 1).

Pregnant women with depressive symptoms are recruited by an advertisement shared on social media by the women’s clinic of Turku University Hospital and by brochures distributed at routine ultrasound scans. The advertisement includes a link to an electronic Edinburgh Pre-/Postnatal Depression Scale (EPDS)-questionnaire. The research team’s doctoral student contacts by phones eligible women, who have provided their contact information, to recruit them.

Those women who agreed to participate in will receive informed consent and the first set of questionnaires by e-mail. During this first contact, the research doctor also schedules the first attachment representation interview with the participants and does a clinical interview to assess the severity of depression. The informed consent forms are signed and collected before starting the first attachment representation interview.

The attachment representation interviews are conducted twice with all the participants between 25 and 35 gwks. Randomization to intervention and follow-up groups occurs after the first attachment representation interview. The participants in the intervention group will receive three interactive ultrasound examinations.

The mother-infant interaction is video-recorded from all participants when the baby is 4–5 months of age.

Background information is collected by means of an electronic questionnaire during the first study visit. Information about depressive and anxiety symptoms, as well as feelings of attachment, is collected by means of electronic questionnaires at three time points: in connection with the attachment representation interviews during pregnancy and the interaction observation visit when the baby is 4–5 months old. The information about the participants’ previous psychiatric diagnoses and pregnancy data are collected from electronic medical records. Prenatal care continues as usual.

## Methods: participants, interventions, and outcomes

### Study setting {9}

In Finland, over 99% of all pregnancies are monitored in Healthy Maternity Clinics [63] that are part of the basic healthcare arranged by the municipalities. The clinics provide screening for fetal chromosomal abnormalities at the end of the first trimester and for severe structural abnormalities between 19 and 21 gwks, as well as offer family support, with attention to relationships and parenting. Risk pregnancies are also referred to maternity outpatient clinics in hospitals. Deliveries and conditions requiring emergency care are treated in hospitals as well. The Finnish health care system supports women experiencing miscarriage, stillbirth, or adverse pregnancy outcomes [64]. The perinatal mortality rate in Finland was 0.4% in 2019 [65].

This study is carried out at the Hospital District of Southwest Finland, at the maternity clinic of Turku University Hospital, and in collaboration with the Department of Psychology, University of Turku. This tertiary level hospital offers extensive specialized health care services to the residents. There are approximately 4000 births annually; in 2020, there were 3811 births [66].

### Eligibility criteria {10}

The inclusion criteria are as follows:(1) 10–15 points on the EPDS, (2) singleton pregnancy, (3) over 18 years of age, (4) body mass index under 35, and (4) fluency in the Finnish language.

The exclusion criteria are psychotic and self-destructive symptoms. The use of antidepressant medication is not an obstacle to participation.

The women are recruited after the routine screening for severe structural abnormalities between 19 and 21 gwks offered by the municipalities. Severe structural abnormalities are an exclusion criterion.

### Who will take informed consent? {26a}

An information letter, which can be found on the project’s webpage is emailed to the women who have completed the depression screener through the webpage. The graduate students in psychology collect the informed consent forms from the participants at the beginning of the first study visit, which is the attachment representation interview.

### Additional consent provisions for collection and use of participant data and biological specimens {26b}

These are not applicable. No biological specimens are collected in this study.

## Interventions

### Explanation for the choice of comparators {6b}

The Finnish Institution for Health and Welfare (THL) recommends that the Healthy Mother Clinics screen for depressive symptoms using EPDS at 13-18 gwks. If the EPDS score is 13 or more, THL recommends that the pregnant woman should be referred to the general practitioner in a Healthy Mother Clinics for further assessment. If the total score is 10–12 points, the EPDS should be repeated within 2-4 weeks. In case of self-destructive thoughts, immediate help should be organized, also on call [67]. All the pregnant women are screened for depressive symptoms using the EPDS in the communal Healthy Mother Clinics. The pregnant woman’s mood should also be assessed by observation and discussion [68].

### Intervention description {11a}

The method is pregnancy ultrasound combined with an interactive approach; a joint observation of the fetus based on the woman’s wishes. An obstetrician, a specialist in maternal-fetal medicine, and a psychologist specialized in psychotherapy and infant mental health jointly conduct the intervention. Three intervention visits are carried out between 25 and 33 gwks because the visibility of the fetus decreases after 33 gwks.

The focus of the ultrasound examination is to follow the fetal activities inside the womb, to work with mental images regarding the fetus and the pregnancy, and listen to the woman expressing her thoughts regarding the fetus. In the beginning, the obstetrician ensures that the pregnant woman understands the images on the screen. Thereafter, the obstetrician, the psychologist, and the woman jointly watch what the fetus does during the examination. Observations are made on what is seen on the screen and on fetal activities taking into account the woman’s reactions. The sonographer and the psychologist avoid making any interpretations about the fetus or its behavior. Rather, they comment on the behavior as it is happening. The aims are to facilitate and emphasize pregnant women’s own perceptions of the fetus. With 4D ultrasound, it is possible to see the fetus’ face and facial expressions and discuss these with the mother. The intervention visits follow a structured course (Appendix 2. The course of intervention visits). The examination lasts approximately 40 min, and the women participate alone without the presence of the fetus’ father. The ultrasound examinations are videotaped for later analysis.

The other parent may join the ultrasound examination once after the actual intervention to see the fetus.

### Criteria for discontinuing or modifying allocated interventions {11b}

No modifications have been made so far. All participants receive the same intervention treatment; three interactive ultrasound examinations lasting approximately 40 min each. In case of a pregnancy complication, the woman is referred to a clinical check-up. Also, in case of fetal demise, an obstetrician is contacted immediately. Interactive ultrasound examinations are conducted in the same outpatient clinic where risk pregnancies are monitored. The obstetricians, who are specialists in maternal-fetal medicine are also employees of the women’s clinic in Turku University Hospital.

### Strategies to improve adherence to interventions {11c}

The three intervention teams each comprise a psychologist and an obstetrician. All interventionists receive training in the interactive approach. The structure of each session is described, and key themes of the sessions are listed. Intervention teams meet together frequently to review the video-recorded intervention sessions in order to enhance adherence to the course of the intervention visits.

### Relevant concomitant care permitted or prohibited during the trial {11d}

Prenatal care continues as usual. Other support or therapeutic treatments are also allowed.

### Provisions for post-trial care {30}

No provisions for post-trial care are necessary.

### Outcomes {12}

The primary outcome of the study is perinatal depressive symptoms analyzed as a change in the EPDS- mean score from before the intervention to after the intervention, maternal attachment, and prenatal representations before and after the intervention. The secondary outcomes are maternal-infant-relationship- related outcomes; maternal pregnancy-related psychological distress, and mother-infant interaction. The secondary outcomes are analyzed with both quantitative and qualitative methods.

### Participant timeline {13}

The participant timeline is described in Appendix 1. which is a research chart.

### Sample size {14}

The study aims to compare changes in EPDS scores between two independent groups. A mean decrease of three points in the EPDS score is considered clinically relevant and used for power calculations. A standard deviation (SD) of 3.5 points in the EPDS score used in power calculations was based on earlier studies in the Finnish population showing an SD of 3.7–4 [25]. A power analysis using a statistical power of 80% and a significance level of 0.05 (two-tailed) showed that 50 subjects are needed in both groups. Because of an estimated attrition rate of 10%, 60 subjects for both groups are currently being enrolled. Randomization was performed with random permuted blocks, using block size 10. The SAS® was used to generate the randomization (Version 9.4 for Windows).

### Recruitment {15}

Pregnant women with depressive symptoms are recruited by an advertisement repeatedly shared in social media by the Department of Obstetrics and Gynecology in Turku University Hospital and by brochures distributed during routine ultrasound scans. The recruitment advertisement includes a link to the research project’s web page, where the pregnant women can fill out an electronic EPDS questionnaire and provide their contact information. The research team’s doctoral student phones those women who have received 10-15 points on the EPDS questionnaire in order to recruit them. During the first contact, the doctoral student ensures that the women meet the inclusion criteria, interviews them with Structured Clinical Interview for Diagnostic and Statistical Manual of Mental Disorders (DSM)-IV-TR (SCID), and makes an appointment for the first attachment representation interview (Working Model of the Child Interview [WMCI]). The doctoral student also contacts the women who have received 16 or more on the EPDS questionnaire or reported suicidal thoughts in order to identify their need for support.

Due to the COVID-19 pandemic, the recruitment was suspended from March 16 to September 22, 2020, but has continued since then.

## Assignment of interventions: allocation

### Sequence generation {16a}

Randomization for the intervention and the control groups was performed with random permuted blocks, using block size 10. SAS® was used to generate the randomization (Version 9.4 for Windows, SAS Institute Inc., Cary, NC, USA), and programming was done by a biostatistician.

### Concealment mechanism {16b}

The randomization list is kept in a locked place to which only the study group has access. After the first attachment representation interview, the interviewer looks at a separate randomization list to find out the group to which the participant is assigned and reveals it to the participant. Two graduate students of psychology carry out the interviews, which are scheduled according to the gestational weeks of the participants.

### Implementation {16c}

A biostatistician carried out the allocation sequence before the start of the present randomized study. The doctoral student contacts potentially eligible women to recruit them, ensures that they meet the inclusion criteria, conducts an SCID interview, and makes an appointment for the first attachment representation interview. The graduate students of psychology conduct the first WMCI- interview, they then look at the allocation sequence to find out the group the participant is assigned. Two graduate students work on the project simultaneously and have received training to follow the study protocol.

## Assignment of interventions: blinding

### Who will be blinded {17a}

Blinding of the coders of the representation interviews and the mother-infant interaction is important for avoiding bias. However, two coders who rate the attachment representation interviews are not completely blinded, because one of them is a member of the intervention team. To avoid bias, the coder who rates the second representation interview does not know the group to which the participant belongs. However, the participants may talk about the intervention visits during their post-intervention interview, thus revealing their group allocation.

Similarly, the coders of the mother-infant interaction situations are not completely blinded. Oner is the main coder, doing 100% of the codings, and is not completely blinded to the sample because she has interviewed some participants and may thus know their study group status. However, the reliability coder, who does 25% of the codings overlapping those done by the main coder, is completely blinded for the group status.

The participants know to which group they belong, which is also a potential source of bias. However, in the informed consent form, they are not exactly told about the primary outcomes of the study.

### Procedure for unblinding if needed {17b}

This is not applicable. See the “Who will be blinded {17a}” section.

## Data collection and management

### Plans for assessment and collection of outcomes {18a}

The study’s outcomes are assessed and collected using a mixed-methods approach that comprises self-reported and observation-based measurements. The data on the self-reported measurements are collected using electronic questionnaires. The data on the observation-based assessments are collected from video-recorded interviews and interaction situations. Because of the COVID-19-pandemic, the interviews may be carried out with remote connections.

The data from the questionnaires are collected and managed using the Research Electronic Data Capture (REDCap) tools hosted in the University of Turku [69, 70]. REDCap is a secure, web-based software platform designed to support data capture for research studies, providing (1) an intuitive interface for validated data capture, (2) audit trails for tracking data manipulation and export procedures, (3) automated export procedures for seamless data downloads to common statistical packages, and (4) procedures for data integration and interoperability with external sources.

#### Data collection measures

The women’s background variables’ such as parity, age, antidepressant medication, and care contacts within healthcare are collected electronically by REDCap in connection with other questionnaires before the first attachment representation interview.

#### Prenatal and postnatal depressive symptoms

Prenatal and postnatal depressive symptoms are assessed using the Edinburgh Pre-/Postnatal Depression Scale (EPDS). The EPDS is a reliable, validated, and internationally accepted 10-item self-rating instrument for screening depressive symptoms, with the score ranging from 0 to 30 points [7, 71]. For minor depression, the lowest cut-off score is 10 points [7, 71, 72]. The cut-off score for major depression varies from 13 points [73, 74] to 15 points [74]. In the Finnish cohort, the cut-off score of ≥ 12 points is used for probable depression [75]. The higher the score, the higher the probability of clinical depression.

The EPDS questionnaire is filled out electronically by REDCap at three time points: in connection with the two attachment representation interviews and the interaction observation visit in the postpartum period. During the recruitment phase, the first EPDS questionnaire is filled out voluntarily on the project’s web page by those women who are interested in participating. After their recruitment, the participants individually receive the links to the electronic questionnaires.

#### Depression

Depressive symptoms are also evaluated by The Structured Clinical Interview for Diagnostic and Statistical Manual of Mental Disorders (SCID). It is a valid, diagnostic, and widely used semi-structured interview designed to evaluate psychopathology categorically. For example, it can be used to assess the severity of dimensions of depression [76]. The interview is reliable in clinical screening as well as for research purposes and can be conducted over the phone [77]. Only the depression module of the SCID is used in the present study. The SCID is chosen to verify clinical depression and its severity.

Using the SCID, the doctoral student conducts phone interviews with eligible women upon recruitment.

#### Pregnancy-Related Anxiety

Pregnancy-specific anxiety is assessed with the Pregnancy-Related Anxiety Questionnaire Revised (PRAQ -R2) [78, 79], which is a validated and shortened version of the 34-item PRAQ [80]. PRAQ-R2 is suitable for use in pregnant women regardless of parity. It is a 10-item self-report scale that consists of three subscales: (1) fear of giving birth, (2) fear of bearing children with physical or mental handicapped, and (3) concern about one’s appearance. Each item score ranges from 1 (definitely not true) to 5 (definitely true). A higher score represents a higher level of anxiety.

The PRAQ-R2 is filled out electronically by REDCap twice in connection with the representation interviews. After their recruitment, the participants individually receive the links to electronic questionnaires.

#### General anxiety

General anxiety symptoms are measured with the anxiety subscale of the Symptoms Checklist 90 (SCL-90), which is a valid and reliable 10-item self-report, with each item rated from 0 (not at all) to 4 (extremely) [81]. It can be used in both clinical and research settings [82].

The anxiety subscale of the SCL-90 questionnaire is filled out electronically by REDCap twice, in connection with the representation interviews. After their recruitment, the participants individually receive the links to the electronic questionnaires.

#### Prenatal maternal relationship with the infant

##### Prenatal attachment

Prenatal maternal emotional attachment is measured using the Maternal Antenatal Attachment Scale (MAAS), which is a validated 19-item instrument designed to evaluate prenatal attachment to the fetus using the dimensions of “quality of attachment” (10 items e.g. experiences of closeness, tenderness, pleasure in interaction, distress at fantasized loss and conceptualizing the fetus as a “little person”) and “intensity/time spent in attachment mode” (8 items, e.g., the amount of time spent thinking about, talking to, dreaming about or palpating the fetus, quality of involvement and intensity of preoccupation). One item (feeling whether the fetus is dependent on the mother for its well-being) is excluded from either dimension but is included in the global score. A high combined score indicates a strong attachment to and relationship with the fetus [18, 83, 84].

The MAAS questionnaire is filled out electronically by REDCap twice in connection with the representation interviews. After their recruitment, the participants individually receive the links to the electronic questionnaires.

##### Attachment representations

Prenatal attachment representations are assessed using the Working Model of the Child Interview (WMCI) [85]. This semi-structured interview systematically explores the parents’ thoughts, feelings, and perceptions at the moment and in the future, about their fetus and relationship with their expected baby. This study uses a prenatal version of the attachment representation interview [28]. The interviews are conducted and recorded in either a laboratory or a home setting. Because of COVID-19 restrictions, remote interviews are also allowed, which are conducted with safe online conferencing and recording software. The interviews are carried out by the graduate students in psychology, who are trained in the interview protocol.

From the recorded interviews, representation narratives are evaluated according to the WMCI coding manual [86]. First, the narrative is evaluated with six qualitative scales (Richness of Perceptions, Openness to Change, Intensity of Involvement, Coherence, Caregiving Sensitivity, and Acceptance) and two content scales (Infant Difficulty and Fear of Infant’s Safety). The quality and content scales are coded with a Likert-type scale ranging from 1 to 5 [86]. Subsequently, the narratives are categorized into balanced, disengaged, and distorted representations. Disengaged and distorted representations can also be classified as non-balanced representations.

Two trained coders who have attained an appropriate level of reliability in their training, with more than 80% agreement between the trainer and the trainee, analyze the attachment representation interviews.

The graduate students in psychology conduct the representation interviews twice at gestational weeks 25 and at 33–35.

#### Postnatal mother-infant relationship

##### Postnatal attachment

Postnatal maternal emotional attachment is measured using the Maternal Postnatal Attachment Scale (MPAS), which is a validated 19-item postpartum counterpart of Condon’s MAAS [84]. It measures the mother’s reported feelings about her infant by three factors: (1) quality of bonding, (2) absence of hostility, and (3) pleasure in interaction [87]. A higher score indicates a more adaptive mother-infant bonding style [87].

The MPAS questionnaire is filled out electronically by REDCap in connection with the postpartum interaction observation visit. After their recruitment, the participants individually receive the links to the electronic questionnaires.

##### Mother-infant interaction

The quality of the mother-infant interaction is assessed using the Parent-Child Early Relational Assessment (PCERA) [88, 89], a video-observation method. The interaction situations are recorded by the graduate students in psychology 4–5 months postpartum. In the current study, the mother-infant interaction is video-recorded during a free-play session using a standardized set of toys. The videos are recorded in a laboratory setting to standardize the environment. The situations are analyzed according to the PCERA manual [89], which consists of 65 independent items: 29 parental, 28 children, and eight dyadic items. Ratings are based on a 5-min interactions sequence. The items are scored from 1 to 5; scores 1 and 2 represent an area of concern, 3 denotes an area of some concern, and 4 and 5 signify an area of strength. Two trained coders who have achieved an appropriate level of reliability, with more than 80% agreement with a trainer of the method, analyze the quality of the interaction according to the manual. The method has good factorial validity and evidence for convergent and discriminant validity as well [88, 90].

### Plans to promote participant retention and complete follow-up {18b}

Participant retention can be strengthened by phone calls or short message service as needed.

### Data management {19}

A data file description is required by the Ethics Committee of the Hospital District of Southwest Finland in order to obtain research permission. The participants’ files are preserved separately from the research data in a secure place. The participants are assigned ID codes that will be used for data storage and analysis. The research data are pseudoanonymized: the recruitment log of the participants is used to undo the ID codes and is kept separately from the data files. The form data are stored in REDCap [70], which, in turn, is stored in a secure network drive of the University of Turku. The videotapes are stored in a secure online storage of the University of Turku.

The University of Turku automatically provides copies of the research data into three independent servers when the data are stored. The servers also function as a data backup system. Only the members of the study group have access to the data. Access to the storage is controlled by the principal investigator of the study. Access also requires a personal username issued by the University of Turku. The allowed access to the data is issued only for the active research period. The principal investigator keeps a record of those who have active access to the data. All the data will be disposed of 10 years after the last publication.

### Confidentiality {27}

See the “Data management {19}” section.

### Plans for collection, laboratory evaluation, and storage of biological specimens for genetic or molecular analysis in this trial/future use {33}.

These are not applicable. No biological specimens are collected in this study.

## Statistical methods

### Statistical methods for primary and secondary outcomes {20a}

The categorical variables of the study are characterized using frequencies and percentages. Continuous variables with means and SDs or medians with ranges and/or 25th and 75th percentiles are used. Age is summarized with the mean and the range.

If the assumptions for parametric analyses are met, the mean change of the EPDS score over time is analyzed using a linear mixed modeling approach for repeated measures. Then, the time point is considered as a within-factor and the treatment intervention as a between factor-effect. The effects of the participants’ background variables’ parity, age, antidepressant medication, care contacts within healthcare, and so forth, are also tested. The time point x intervention interaction describes whether the mean changes over time differ between the groups. The covariance structure of the unstructured (UN) and the compound symmetry (CS) is tested. The differences (together with 95% confidence interval [CI]) between the groups are also estimated at each time point, as well as within-group changes. Transformation for the EPDS or for other outcomes can be performed before analyses, if needed. The assumptions of parametric tests are checked using studentized residuals for justification of the analyses. Other numerical variables, mean change of MAAS, MPAS, SCL-90, and PRAQ-R2 score over time, are analyzed in a similar way.

*P*-values less than 0.05 are considered statistically significant (two-tailed). Statistical analyses are performed using SAS System for Windows, Version 9.4 or later (SAS Institute Inc., Cary, NC, USA).

### Interim analyses {21b}

These are not applicable. No interim analyses will be done in this study.

### Methods for additional analyses (e.g., subgroup analyses) {20b}

The sample size and the subgroups are small. Preliminary analyses are conducted to find out if there are statistically significant differences within and between the study groups.

### Methods in analysis to handle protocol non-adherence and any statistical methods to handle missing data {20c}

Because of the relatively small sample size, imputation will not be carried out.

### Plans to give access to the full protocol, participant-level data and statistical code {31c}

Metadata can be published on the project’s web-page without compromising the participants’ data protection. The metadata includes information on what kind of data has been collected, on what date, from whom, and how. Requests for collaboration are encouraged.

## Oversight and monitoring

### Composition of the coordinating center and trial steering committee {5d}

This is not applicable. The authors of the protocol coordinate this study.

### Composition of the data monitoring committee, its role and reporting structure {21a}

The study group is independent.

### Adverse event reporting and harms {22}

The doctoral student of the research team continuously follows up on the information collected from the questionnaires and contacts the women who have admitted to having self-destructive thoughts or plans or whose score is ≥ 16 on the EPDS. She asks if they have psychiatric or psychological support or whether they have discussed the issue in the Healthy Maternity Clinic. All the women are told about support providers verbally by phone and with a care instruction letter by e-mail. All the women whose EPDS scores are between 10 and 15 are also contacted because they are recruited for the study.

### Frequency and plans for auditing trial conduct {23}

The study group meets regularly to maintain the consistency of the intervention delivery and the interviews. The intervention visits are videotaped, and the intervention delivery is monitored from the video recordings of the meetings of the interventionists. Separate supervisions are organized for the graduate students of psychology to maintain the consistency of the interview technique and to receive support.

### Plans for communicating important protocol amendments to relevant parties (e.g., trial participants, ethical committees) {25}

Any changes in the study protocol must be accepted by the Ethics Committee of the Hospital District of Southwest Finland before enforcement.

### Dissemination plans {31a}

The results will be published in an international open access publication and will be linked to the project’s web page, with a plain-language summary in Finnish. The questionnaire results or the interview analyses will not be reported to the participants personally.

## Discussion

Nonmedical and early relationship-focused therapies should be available to treat prenatal depression because supporting the mother-infant relationship makes it possible to decrease depressive symptoms, improve the subsequent development of the child, and avoid the negative effects of antidepressant medication on infants’ health [13, 34, 35, 43–46]. Psychotherapeutic and psychosocial interventions are effective in treating perinatal depression [91] but require special expertise and resources. Moreover, the reduction in a maternal depressive symptoms alone may not improve parenting or the infant’s well-being [38–41]. Because fetal ultrasound has been considered a promising method to support maternal-fetal attachment [92], an ultrasound-assisted intervention could be a low-threshold type of support and easily feasible in maternity care. Therefore, an ultrasound examination used in an interactive manner may be a potential and cost-effective way to support pregnant women with minor or moderate depressive symptoms.

The strengths of our study are the mixed methods and the longitudinal approach that continues into the postpartum period. Furthermore, the multiprofessional approach enables the support of pregnant women in various ways. A weakness in our randomized controlled trial may be the control group’s participation in the attachment representation interviews, which can be experienced as an intervention itself.

Our aim is to develop an intervention for pregnant women with depressive symptoms using 4D ultrasound in an interactive way. The interactive approach is unique and could be integrated into maternity care with a moderate investment. The core elements of the intervention listening to the mother, personalizing the fetus, and giving emotional support can easily be implemented during routine screening and maternity clinic visits. This intervention could serve as a means for maternity care to help women suffering from minor perinatal depression.

## Trial

The protocol version number is five as of December 7, 2021. The first participant was recruited on September 19, 2018. By December 7, 2021, 66 participants have been recruited. The recruitment will be completed within 2 years.

## Figures and Tables

**Fig. 1:**
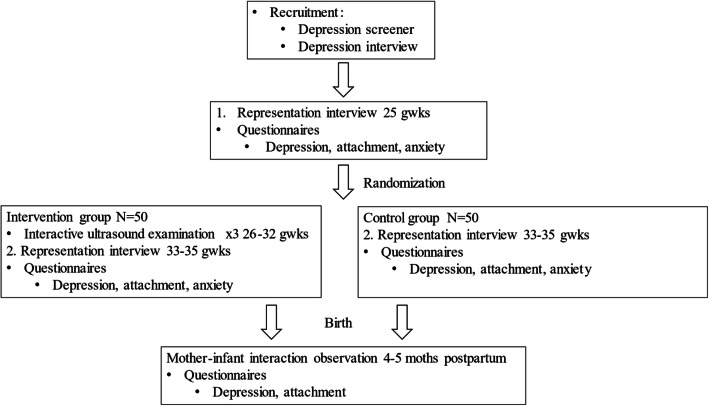
Research chart

## Data Availability

The metadata will be published on the project’s web page.

## Appendix 2

The course of the first intervention visit

1. The beginning:
  - Ask, how has your (both fetus and pregnant woman) day been?
  - Ask, when has the woman seen her fetus last?
  - Ask for the wishes regarding the ultrasound examination: Is there something special, that you would like us to look at together?
2. Viewing together with the pregnant woman:
  - The gynecologist doing the ultrasound explains to the woman what is seen on the screen and makes sure, that the woman perceives the picture of the baby well enough.
  - Observing together the movements of the fetus’s hands and feet, mouth (sucking, licking, swallowing), and fetus’s face and facial expressions.
  - In general, the fetus is discussed as an individual person.
  - The gynecologist tells about the observations only in a behavioral level (e.g., fetus puts her/his hand on the cheek – not that fetus is described as thoughtful). This way the mother has been given space to create her own meanings to the fetus’s behavior.
  - Fetus’s position and the position of limbs can be explored.
  - Sensation of the movements can be asked, when the fetus’s movements are seen on the screen.
  - It is possible to ask what the mother thinks the fetus is experiencing or feeling.
  - To ask what feelings and emotions looking at the fetus rise.
3. The end
  - Is there something that you would like us to look at together?
  - How has it felt to look at your fetus this way?
  - In the end, the gynecologist doing the ultrasound explains without medical terminology to the woman/mother/parents her perception of the fetus’s well-being.

The course of the second intervention visit

1. The beginning
  - Ask, how has your (both fetus and pregnant woman) day been?
  - Ask, what kind of thought did arise after the last visit?
  - Ask, has the woman seen her fetus in ultrasound examination between the visits?
  - Ask, how has the pregnancy gone?
  - Ask for the woman’s wishes regarding the viewing, is there something special, that you would like us to look at together?
2. Viewing together with the pregnant woman
  - Observing together the movements of the fetus’s hands and feet, mouth (sucking, licking, swallowing), and fetus’s face and facial expressions.
  - Observation only in the behavioral level.
  - Discuss together, how the fetus’s behavior is similar or different compared to last visit.
  - Observation of fetus’s position and the location of fetus’s body parts in the womb.
  - Ask the woman/mother if she feels the movements when the fetus is visibly in motion in the ultrasound.
  - Focusing more to questions regarding what the pregnant woman thinks her fetus is experiencing or feeling. Ask, what makes you think so?
  - Sometimes you can ask the woman to tell about the feelings that looking the fetus arises.
    - Thoughts about fetus’s personality?
    - Especially, if looking arises negative emotions/thoughts, ask the woman what makes her think that way.
  - If the woman is worried about something, the worry is listened to and reacted to. It is also important to tell the woman, if the answer is not known.
  - Pregnant woman’s positive observations will always be shared.
3. The end
  - Is there still something that you would like us to look at together?
  - How did it feel to look at the fetus?
  - In the end, the gynecologist doing the ultrasound explains without medical terminology to the woman her perception of the fetus’s well-being.

The course of the third intervention visit

1. The beginning:
  - Ask, how has your (both fetus and pregnant woman) day been?
  - Ask, what kind of thoughts did arise after the last visit?
  - Ask, has the woman seen her fetus in ultrasound examination between the visits?
  - Ask, how has the pregnancy gone?
  - Ask for the woman’s wishes regarding the viewing, is there something special, that you would like us to look at together?
2. Viewing together with the pregnant woman:
  - Observing together the movements of the fetus’s hands and feet, mouth (sucking, licking, swallowing), and fetus’s face and facial expressions.
  - Observation only in the behavioral level.
  - Discuss together, how the fetus’s behavior is similar or different compared to the last visit.
  - Observation of fetus’s position and the location of fetus’s body parts in the womb.
  - Ask the pregnant woman if she feels the movements when the fetus is visibly in motion in the ultrasound.
  - Sometimes you can ask what the pregnant woman thinks the fetus is experiencing or feeling.
  - Sometimes you can ask the woman to tell about the feelings that looking at the fetus arises.
  - If the pregnant woman is worried about something, the worry is listened and reacted to. It is also important to tell the woman, if the answer is not known.
  - Pregnant woman’s positive observations will always be shared.
  - It is possible to reflect together on woman’s thoughts about the upcoming childbirth
    - When thinking about pregnancy onwards, how does it feel?
    - What kind of birth do you wish for your fetus and yourself?
    - How are you expecting seeing your fetus?
    - How is the fetus right after delivery?
    - Has looking at the fetus with ultrasound invited any reflections about how is the fetus going to be like? If the mother describes the fetus, it is possible to ask how do the characteristics feel.
3. The end
  - Is there still something that you would like us to look at together?
  - How have you experienced these visits?
  - In the end, the gynecologist doing the ultrasound explains without medical terminology to the woman her perception of the fetus’s well-being.

## Abbreviations

4D: Four dimensional
3D: Three dimensional
EPDS: Edinburgh Pre-/Postnatal Depression Scale
WMCI: Working Model of the Child Interview
PCERA: Parent-Child Early Relational Assessment
MAAS: Maternal Antenatal Attachment Scale
MPAS: Maternal Postnatal Attachment Scale
PRAQ-R2: Pregnancy-Related Anxiety Questionnaire Revised
SCL-90: Symptoms Checklist 90
SCID: Structured Clinical Interview for DSM
DSM: Diagnostic and Statistical Manual of Mental Disorders
IUGR: Intrauterine growth restriction
gwks: Gestational weeks
THL: Finnish Institution for Health and Welfare
REDCAp: Research Electronic Data Capture
SSRIs: Selective serotonin reuptake inhibitors
SD: Standard deviation
UN: Covariance structure of the unstructured
CS: Compound symmetry
CI: Confidence interval

## References

[1] Woody CA, Ferrari AJ, Siskind DJ, Whiteford HA, Harris MG. A systematic review and meta-regression of the prevalence and incidence of perinatal depression. J Affective Disord. 2017. 10.1016/j.jad.2017.05.003. 10.1016/j.jad.2017.05.003 28531848

[2] Gavin NI, Gaynes BN, Lohr KN, Meltzer-Brody S, Gartlehner G, Swinson T. Perinatal depression: A systematic review of prevalence and incidence. Obstet Gynecol. 2005. 10.1097/01.AOG.0000183597.31630.db. 10.1097/01.AOG.0000183597.31630.db 16260528

[3] Tammentie T, Tarkka MT, Åstedt-Kurk P, Paavilainen E. Sociodemographic factors of families related to postnatal depressive symptoms of mothers. Int J Nurs Pract. 2002. 10.1046/j.1440-172x.2002.00373.x. 10.1046/j.1440-172x.2002.00373.x 12225350

[4] Pajulo M, Savonlahti E, Sourander A, Helenius H, Piha J. Antenatal depression, substance dependency and social support. J Affect Disord. 2001. 10.1016/s0165-0327(00)00265-2. 10.1016/s0165-0327(00)00265-2 11426516

[5] Ahlqvist-Björkroth S, Vaarno J, Junttila N, Pajulo M, Räihä H, Niinikoski H, et al. Initiation and exclusivity of breastfeeding: Association with mothers’ and fathers’ prenatal and postnatal depression and marital distress. Acta Obstet Gynecol Scand. 2016. 10.1111/aogs.12857. 10.1111/aogs.12857 26826608

[6] Gaynes BN, Gavin N, Meltzer-Brody S, Lohr KN, Swinson T, Gartlehner G, et al. Perinatal depression: prevalence, screening accuracy, and screening outcomes. Evidence Report/Technol Assess (Summary). 2005. 10.1037/e439372005-001. 10.1037/e439372005-001 PMC4780910 15760246

[7] Bergink V, Kooistra L, Lambregtse-van den Berg MP, Wijnen H, Bunevicius R, van Baar A, et al. Validation of the Edinburgh Depression Scale during pregnancy. J Psychosom Res. 2011. 10.1016/j.jpsychores.2010.07.008. 10.1016/j.jpsychores.2010.07.008 21414460

[8] Korja R, Nolvi S, Kataja EL, Scheinin N, Junttila N, Lahtinen H, et al. The courses of maternal and paternal depressive and anxiety symptoms during the prenatal period in the finnbrain birth cohort study. PLoS One. 2018. 10.1371/journal.pone.0207856. 10.1371/journal.pone.0207856 PMC6296666 30557345

[9] Ashley JM, Harper BD, Arms-Chavez CJ, LoBello SG. Estimated prevalence of antenatal depression in the US population. Arch Womens Ment Health. 2016. 10.1007/s00737-015-0593-1. 10.1007/s00737-015-0593-1 26687691

[10] Marchesi C, Bertoni S, Maggini C. Major and minor depression in pregnancy. Obstet Gynecol. 2009. 10.1097/AOG.0b013e3181a45e90. 10.1097/AOG.0b013e3181a45e90 19461425

[11] Eastwood J, Ogbo FA, Hendry A, Noble J, Page A. The impact of antenatal depression on perinatal outcomes in Australian women. PLoS One. 2017. 10.1371/journal.pone.0169907. 10.1371/journal.pone.0169907 PMC5241141 28095461

[12] Ogbo FA, Eastwood J, Hendry A, Jalaludin B, Agho KE, Barnett B, et al. Determinants of antenatal depression and postnatal depression in Australia. BMC Psychiatry. 2018. 10.1186/s12888-018-1598-x. 10.1186/s12888-018-1598-x PMC5819705 29463221

[13] Goecke TW, Voigt F, Faschingbauer F, Spangler G, Beckmann MW, Beetz A (2012). The association of prenatal attachment and perinatal factors with pre- and postpartum depression in first-time mothers. Arch Gynecol Obstet..

[14] Leigh B, Milgrom J. Risk factors for antenatal depression, postnatal depression and parenting stress. BMC Psychiatry. 2008. 10.1186/1471-244X-8-24. 10.1186/1471-244X-8-24 PMC2375874 18412979

[15] Underwood L, Waldie K, D’Souza S, Peterson ER, Morton S. A review of longitudinal studies on antenatal and postnatal depression. Arch of Women’s Ment. Health. 2016. 10.1007/s00737-016-0629-1. 10.1007/s00737-016-0629-1 27085795

[16] McFarland J, Salisbury AL, Battle CL, Hawes K, Halloran K, Lester BM. Major depressive disorder during pregnancy and emotional attachment to the fetus. Arch Womens Ment Health. 2011. 10.1007/s00737-011-0237-z. 10.1007/s00737-011-0237-z PMC3248759 21938509

[17] Seimyr L, Sjögren B, Welles-Nyström B, Nissen E. Antenatal maternal depressive mood and parental-fetal attachment at the end of pregnancy. Arch Womens Ment Health. 2009. 10.1007/s00737-009-0079-0. 10.1007/s00737-009-0079-0 19499285

[18] Condon JT, Corkindale C. The correlates of antenatal attachment in pregnant women. Br J Med Psychol. 1997. 10.1111/j.2044-8341.1997.tb01912.x. 10.1111/j.2044-8341.1997.tb01912.x 9429755

[19] Rubertsson C, Pallant JF, Sydsjö G, Haines HM, Hildingsson I (2015). Maternal depressive symptoms have a negative impact on prenatal attachment – findings from a Swedish community sample. J Reprod Infant Psychol..

[20] Siddiqui A, Hägglöf B (2000). Does maternal prenatal attachment predict postnatal mother-infant interaction?. Early Hum Dev..

[21] Dubber S, Reck C, Müller M, Gawlik S. Postpartum bonding: the role of perinatal depression, anxiety and maternal–fetal bonding during pregnancy. Arch Womens Ment Health. 2015;18(2):187–95. 10.1007/s00737-014-0445-4 25088531

[22] Cuijlits I, van de Wetering AP, Endendijk JJ, van Baar AL, Potharst ES, Pop VJM. Risk and protective factors for pre- and postnatal bonding. Infant Ment Health J. 2019. 10.1002/imhj.21811 31430393

[23] Dayton CJ, Levendosky AA, Davidson WS, Bogat GA. The child as held in the mind of the mother: The influence of prenatal maternal representations on parenting behaviors. Infant Ment Health J. 2010; 10.1002/imhj.20253 28543330

[24] Tambelli R, Odorisio F, Lucarelli L. Prenatal and postnatal maternal representations in nonrisk and at-risk parenting: Exploring the influences on mother-infant feeding interactions. Infant Ment Health J. 2014; 10.1002/imhj.21448 25798489

[25] Ahlqvist-Björkroth S, Korja R, Junttila N, Savonlahti E, Pajulo M, Räihä H (2016). Mothers’ and fathers’ prenatal representations in relation to marital distress and depressive symptoms. Infant Ment Health J..

[26] Ammaniti M, Tambelli R, Odorisio F. Exploring Maternal Representations During Pregnancy in Normal and At-Risk Samples: The Use of the Interview of Maternal Representations During Pregnancy. Infant Ment Health J. 2013.

[27] Crawford A, Benoit D. Caregivers’ disrupted representations of the unborn child predict later infant-caregiver disorganized attachment and disrupted interactions. Infant Ment Health J. 2009. 10.1002/imhj.20207 28636176

[28] Benoit D, Parker KCH, Zeanah CH. Mothers’ representations of their infants assessed prenatally: Stability and association with infants’ attachment classifications. J Child Psychol Psychiatry Allied Discip. 1997. 10.1111/j.1469-7610.1997.tb01515.x 9232477

[29] Talge NM, Neal C, Glover V. Antenatal maternal stress and long-term effects on child neurodevelopment: How and why? J Child Psychol Psychiatry Allied Disciplines. 2007. 10.1111/j.1469-7610.2006.01714.x PMC11016282 17355398

[30] Van Den Bergh BRH, Mulder EJH, Mennes M, Glover V (2005). Antenatal maternal anxiety and stress and the neurobehavioural development of the fetus and child: Links and possible mechanisms. A review. Neuroscience and Biobehavioral Reviews.

[31] Kingston D, Tough S, Whitfield H. Prenatal and postpartum maternal psychological distress and infant development: A systematic review. Child Psychiatry Hum Dev. 2012. 10.1007/s10578-012-0291-4 22407278

[32] Tuovinen S, Lahti-Pulkkinen M, Girchenko P, Lipsanen J, Lahti J, Heinonen K, et al. Maternal depressive symptoms during and after pregnancy and child developmental milestones. Depress Anxiety. 2018. 10.1002/da.22756 29667739

[33] Malm H, Artama M, Brown AS, Gissler M, Gyllenberg D, Hinkka-Yli-Salomäki S, et al. Infant and childhood neurodevelopmental outcomes following prenatal exposure to selective serotonin reuptake inhibitors: Overview and design of a Finnish Register-Based Study (FinESSI). BMC Psychiatry. 2012. 10.1186/1471-244X-12-217 PMC3564781 23206294

[34] Malm H, Sourander A, Gissler M, Gyllenberg D, Hinkka-Yli-Salomäki S, McKeague IW, et al. Pregnancy complications following prenatal exposure to SSRIs or maternal psychiatric disorders: Results from population-based national register data. Am J Psychiatry. 2015. 10.1176/appi.ajp.2015.14121575 26238606

[35] Malm H, Brown AS, Gissler M, Gyllenberg D, Hinkka-Yli-Salomäki S, McKeague IW, et al. Gestational Exposure to Selective Serotonin Reuptake Inhibitors and Offspring Psychiatric Disorders: A National Register-Based Study. J Am Acad Child Adolesc Psychiatry. 2016. 10.1016/j.jaac.2016.02.013 PMC4851729 27126849

[36] Khanlari S, Barnett Am B, Ogbo FA, Eastwood J. Re-examination of perinatal mental health policy frameworks for women signalling distress on the Edinburgh Postnatal Depression Scale (EPDS) completed during their antenatal booking-in consultation: A call for population health intervention. BMC Pregnancy Childb. 2019. 10.1186/s12884-019-2378-4 PMC6604146 31266468

[37] Freeman MP. Perinatal Depression: Recommendations for Prevention and the Challenges of Implementation. JAMA- J Am Med Assoc. 2019. 10.1001/jama.2018.21247 30747953

[38] Forman DR, O’Hara MW, Stuart S, Gorman LL, Larsen KE, Coy KC. Effective treatment for postpartum depression is not sufficient to improve the developing mother-child relationship. Dev Psychopathol. 2007. 10.1017/S0954579407070289 17459185

[39] Tsivos ZL, Calam R, Sanders MR, Wittkowski A. Interventions for postnatal depression assessing the mother–infant relationship and child developmental outcomes: A systematic review. Int Women’s Health. 2015. 10.2147/IJWH.S75311 PMC4412485 25960678

[40] Nylen KJ, Moran TE, Franklin CL, O’Hara MW. Maternal depression: A review of relevant treatment approaches for mothers and infants. Infant Mental Health J. 2006. 10.1002/imhj.20095 28640416

[41] Poobalan AS, Aucott LS, Ross L, Smith WCS, Helms PJ, Williams JHG. Effects of treating postnatal depression on mother-infant interaction and child development: Systematic review. Br J Psychiatry. 2007. 10.1192/bjp.bp.106.032789 17978316

[42] Clark R, Tluczek A, Brown R. A mother-infant therapy group model for postpartum depression. Infant Ment Health J. 2008. 10.1002/imhj.20189 28636219

[43] Stein A, Pearson RM, Goodman SH, Rapa E, Rahman A, McCallum M, et al. Effects of perinatal mental disorders on the fetus and child. Lancet. 2014; 10.1016/S0140-6736(14)61277-0 25455250

[44] Tichelman E, Westerneng M, Witteveen AB, Van Baar AL, Van Der Horst HE, De Jonge A, et al. Correlates of prenatal and postnatal motherto-infant bonding quality: A systematic review. PLoS One. 2019. 10.1371/journal.pone.0222998 PMC6759162 31550274

[45] Lefkovics E, Baji I, Rigó J. Impact of maternal depression on pregnancies and on early attachment. Infant Ment Health J. 2014. 10.1002/imhj.21450 25798487

[46] Salo SJ, Flykt M, Mäkelä J, Biringen Z, Kalland M, Pajulo M, et al. The effectiveness of Nurture and Play: A mentalisation-based parenting group intervention for prenatally depressed mothers. Prim Heal Care Res Dev. 2019. 10.1017/S1463423619000914 PMC7003526 31839012

[47] Glover V, Capron L. Prenatal parenting. Curr Opin Psychol. 2017. 10.1016/j.copsyc.2017.02.007 28813271

[48] Sedgmen B, McMahon C, Cairns D, Benzie RJ, Woodfield RL (2006). The impact of two-dimensional versus three-dimensional ultrasound exposure on maternal-fetal attachment and maternal health behavior in pregnancy. Ultrasound Obstet Gynecol..

[49] Reading AE, Campbell S, Cox DN, Sledmere CM (1982). Health beliefs and health care behaviour in pregnancy. Psychol Med [Internet]..

[50] de Jong-Pleij EAP, Ribbert LSM, Pistorius LR, Tromp E, Mulder EJH, Bilardo CM (2013). Three-dimensional ultrasound and maternal bonding, a third trimester study and a review. Prenat Diagn..

[51] Yarcheski A, Mahon NE, Yarcheski TJ, Hanks MM, Cannella BL. A meta-analytic study of predictors of maternal-fetal attachment. IntJ Nurs Stud. 2009. 10.1016/j.ijnurstu.2008.10.013 19081091

[52] Wenstrom KD (2007). Preexamination and postexamination assessment of parental-fetal bonding in patients undergoing 3-/4-dimensional obstetric ultrasonography: Commentary. Vol. 62, Obstetrical and Gynecological Survey.

[53] Righetti PL, Dell’Avanzo M, Grigio M, Nicolini U. Maternal/paternal antenatal attachment and fourth-dimensional ultrasound technique: A preliminary report. Br J Psychol. 2005. 10.1348/000712604X15518 15826328

[54] Boukydis Z (2006). Ultrasound consultation to reduce risk and increase resilience in pregnancy. Annals of the New York Academy of Sciences.

[55] Boukydis CFZ, Treadwell MC, Delaney-Black V, Boyes K, King M, Robinson T (2006). Women’s Responses to Ultrasound Examinations During Routine Screens in an Obstetric Clinic. J Ultrasound Med [Internet]..

[56] Pajulo H, Pajulo M, Jussila H, Ekholm E (2016). Substance-abusing pregnant women: prenatal intervention using ultrasound consultation and mentalization to enhance the mother–child relationship and reduce substance use. Infant Ment Health J..

[57] Jussila H, Ekholm E, Pajulo M. A New Parental Mentalization Focused Ultrasound Intervention for Substance Using Pregnant Women. Effect on Self-reported Prenatal Mental Health, Attachment and Mentalization in a Randomized and Controlled Trial. Int J Ment Health Addict. 2020.

[58] Jussila H, Pajulo M, Ekholm E. A Novel 4D Ultrasound Parenting Intervention for Substance Using Pregnant Women in Finland: Participation in Obstetric Care, Fetal Drug Exposure, and Perinatal Outcomes in a Randomized Controlled Trial. Matern Child Health J. 2020. 10.1007/s10995-019-02773-w PMC6957471 31250239

[59] Pulliainen H, Niela-Vilén H, Ekholm E, Ahlqvist-Björkroth S. Experiences of interactive ultrasound examination among women at risk of preterm birth: A qualitative study. BMC Pregnancy Childbirth. 2019. 10.1186/s12884-019-2493-2 PMC6751623 31533655

[60] Walsh TB. Your baby is so happy, active, uncooperative: How prenatal care providers contribute to parents’ mental representations of the baby. Midwifery. 2020. 10.1016/j.midw.2020.102630 32006801

[61] Molander E, Alehagen S, Berterö CM. Routine ultrasound examination during pregnancy: a world of possibilities. Midwifery. 2010. 10.1016/j.midw.2008.04.008 18571818

[62] Ekelin M, Svalenius EC, Larsson AK, Nyberg P, Maršál K, Dykes AK. Parental expectations, experiences and reactions, sense of coherence and grade of anxiety related to routine ultrasound examination with normal findings during pregnancy. Prenat Diagn. 2009. 10.1002/pd.2324 19582763

[63] THL: Äitiysneuvola [Internet]. Available from: https://thl.fi/fi/web/lapset-nuoret-ja-perheet/sote-palvelut/aitiys-ja-lastenneuvola/aitiysneuvola. Accessed 8 Mar.

[64] Ministry of Social Affairs and Health [Internet]. Available from: https://stm.fi/en/maternity-and-child-health-clinics

[65] THL Perinataalitilasto 2019 [Internet]. Available from: https://www.julkari.fi/bitstream/handle/10024/140702/Tr48_20.pdf?sequence=1&isAllowed=y

[66] THL. Perinatal statistics - parturients, delivers and newborns 2020 [Internet]. 2020. Available from: https://thl.fi/fi/tilastot-ja-data/tilastot-aiheittain/seksuaali-ja-lisaantymisterveys/synnyttajat-synnytykset-ja-vastasyntyneet/perinataalitilasto-synnyttajat-synnytykset-ja-vastasyntyneet.

[67] Depression. Current Care Guidelines. Working group set by the Finnish Medical Society Duodecim and the Finnish Psychiatric Association. Helsinki: The Finnish Medical Society Duodecim, 2021 (referred March 11, 2021). Available online at: www.kaypahoito.fi [Internet]. Available from: https://www.kaypahoito.fi/.

[68] Klemetti R, Hakulinen-Viitanen T. Äitiysneuvolaopas. Suosituksia äitiysneuvolatoimintaan. [Internet]. THL, Helsinki, Finland. 2013. p. 1-412. Available from: THL_OPA2013_029_verkko.pdf

[69] Harris PA, Taylor R, Thielke R, Payne J, Gonzalez N, Conde JG. Research electronic data capture (REDCap)-A metadata-driven methodology and workflow process for providing translational research informatics support. J Biomed Inform. 2009. 10.1016/j.jbi.2008.08.010 PMC2700030 18929686

[70] Harris PA, Taylor R, Minor BL, Elliott V, Fernandez M, O’Neal L, et al. The REDCap consortium: Building an international community of software platform partners. J Biomed Inform. 2019. 10.1016/j.jbi.2019.103208 PMC7254481 31078660

[71] Cox JL, Holden JM, Sagovsky R. Detection of Postnatal Depression: Development of the 10-item Edinburgh Postnatal Depression scale. Br J Psychiatry. 1987. 10.1192/bjp.150.6.782 3651732

[72] Bunevicius A, Kusminskas L, Pop VJ, Pedersen CA, Bunevicius R. Screening for antenatal depression with the Edinburgh Depression Scale. J Psychosom Obstet Gynecol. 2009. 10.3109/01674820903230708 19845492

[73] Rubertsson C, Börjesson K, Berglund A, Josefsson A, Sydsjö G. The Swedish validation of Edinburgh Postnatal Depression Scale (EPDS) during pregnancy. Nordic J Psychiatry. 2011. 10.3109/08039488.2011.590606 21728782

[74] Matthey S, Henshaw C, Elliott S, Barnett B. Variability in use of cut-off scores and formats on the Edinburgh Postnatal Depression Scale - Implications for clinical and research practice. Arch Womens Ment Health. 2006. 10.1007/s00737-006-0152-x 17013761

[75] Karlsson L, Tolvanen M, Scheinin NM, Uusitupa HM, Korja R, Ekholm E, et al. Cohort Profile: The FinnBrain Birth Cohort Study (FinnBrain). Int J Epidemiol. 2018. 10.1093/ije/dyx173 29025073

[76] Shankman SA, Funkhouser CJ, Klein DN, Davila J, Lerner D, Hee D. Reliability and validity of severity dimensions of psychopathology assessed using the Structured Clinical Interview for DSM-5 (SCID). Int J Methods Psychiatr Res. 2018. 10.1002/mpr.1590 PMC5834362 29034525

[77] Osório FL, Loureiro SR, Hallak JEC, Machado-de-Sousa JP, Ushirohira JM, Baes CVW, et al. Clinical validity and intrarater and test–retest reliability of the Structured Clinical Interview for DSM-5 – Clinician Version (SCID-5-CV). Psychiatry Clin Neurosci. 2019. 10.1111/pcn.12931 31490607

[78] Huizink AC, Mulder EJH, Robles De Medina PG, Visser GHA, Buitelaar JK. Is pregnancy anxiety a distinctive syndrome? Early Hum Dev. 2004. 10.1016/j.earlhumdev.2004.04.014 15324989

[79] Huizink AC, Delforterie MJ, Scheinin NM, Tolvanen M, Karlsson L, Karlsson H. Adaption of pregnancy anxiety questionnaire–revised for all pregnant women regardless of parity: PRAQ-R2. Arch Womens Ment Health. 2016. 10.1007/s00737-015-0531-2 PMC4728175 25971851

[80] Van den Bergh BR. The influence of maternal emotions during pregnancy on fetal and neonatal behavior. J Prenat Perinat Psychol Heal. 1990.

[81] Derogatis LR, Lipman RS, Covi L. SCL-90: an outpatient psychiatric rating scale--preliminary report. Psychopharmacol Bull. 1973. 4682398

[82] Holi MM, Sammallahti PR, Aalberg VA. A Finnish validation study of the SCL-90. Acta Psychiatr Scand. 1998. 10.1111/j.1600-0447.1998.tb09961.x 9504702

[83] MS C. Development of a tool for the measurement of maternal attachment during pregnancy. Nurs Res. 1981. 6912989

[84] Condon JT. The assessment of antenatal emotional attachment: Development of a questionnaire instrument. Br J Med Psychol. 1993. 10.1111/j.2044-8341.1993.tb01739.x 8353110

[85] Zeanah CH, Benoit D, Hirshberg L, Barton ML, Regan C. Mothers’ representations of their infants are concordant with infant attachment classifications. Dev Issues Psychiatry Psychol. 1994.

[86] Zeanah CH, Benoit D, Barton ML, Hirshberg L. Working model of the child interview coding manual. Unpublished manuscript. 1996.

[87] Condon JT, Corkindale CJ. The assessment of parent-to-infant attachment: Development of a self-report questionnaire instrument. J Reprod Infant Psychol. 1998.

[88] Lotzin A, Lu X, Kriston L, Schiborr J, Musal T, Romer G, et al. Observational Tools for Measuring Parent–Infant Interaction: A Systematic Review. Clin Child Fam Psychol Rev. 2015. 10.1007/s10567-015-0180-z 25837491

[89] Clark R. The parent-child early relational assessment: Instrument and manual. Madison Univ Wisconsin Med Sch Dep. Psychiatry. 1985.

[90] Clark R. The parent-child early relational assessment: A factorial validity study. Educ Psychol Meas. 1999.

[91] Sockol LE, Epperson CN, Barber JP. A meta-analysis of treatments for perinatal depression. Clin Psychol Rev. 2011. 10.1016/j.cpr.2011.03.009 PMC4108991 21545782

[92] Borg Cunen N, Jomeen J, Borg Xuereb R, Poat A. A narrative review of interventions addressing the parental–fetal relationship. Women Birth. 2017. 10.1016/j.wombi.2016.11.005 27884654

